# Persistent estrus signs in a spayed bitch following coexistence of ovarian remnant syndrome and exposure to exogenous estrogen: a case report

**DOI:** 10.1007/s11259-026-11499-4

**Published:** 2026-09-09

**Authors:** Alessia Guerini, Giulia Ballotta, Marco Cunto, Cecilia Paltrinieri, Daniele Zambelli

**Affiliations:** Department of Veterinary Medical Sciences, University of Bologna, Ozzano dell’Emilia (BO), Italy

**Keywords:** Bitch, Persistent estrus, Ovarian remnant syndrome, Exogenous hormones, Accidental drugs exposure

## Abstract

Ovarian remnant syndrome represents the most frequent diagnosis in young bitches that present estrous signs some time after ovariectomy/ovariohysterectomy. However, other differential diagnoses should be considered and ruled out, including exogenous estrogen exposure and hormone-secreting neoplasms. The present paper describes a complex clinical case in which ovarian remnant syndrome associated with bilateral ovarian remnants coexisted with exogenous estrogenic stimulation, highlighting the diagnostic challenges and the importance of considering both endogenous and exogenous causes when evaluating spayed bitches with estrous signs.

## Background

The presence of estrous signs in spayed bitches, including vulvar edema, serosanguineous vulvar discharge, and attraction of males, is usually attributed to ovarian remnant syndrome (ORS), which remains the main reported cause in the veterinary literature (Wallace 1991. However, other differential diagnoses should be considered. These include hormone-secreting neoplasms and their metastases. To date, however, the available evidence is limited to estrogen-secreting adrenal tumors described in cats (Meler et al. 2011; Nadolski et al. 2016), with no reports of comparable tumors in dogs. Likewise, no evidence of hormonally active metastatic lesions arising from these tumors has been reported in either species. Another important differential diagnosis is exposure to exogenous estrogens used by pet owners for hormone replacement therapy (Kopper et al. 2009; Carroll 2010). These substances are widely used in human medicine in various formulations, particularly for the management of menopausal symptoms, which increases the likelihood of unintentional exposure in household pets. Accidental contact with these drugs may induce estrogenic stimulation and consequently mimic estrous signs (Wiener et al. 2015; Sterman et al. 2019; Ganz and Wehrend 2021; Ivaldi et al. 2022; May et al. 2024).

Although less commonly encountered, clinical signs of estrogenic stimulation may also result from iatrogenic administration of estrogen-based compounds (e.g. estriol), occasionally used in veterinary medicine for the management of urinary incontinence in spayed bitches (Reichler and Hubler 2014). Such treatments can induce clinical or haematological signs of persistent estrus that may confound diagnostic interpretation (Barsanti et al. 1983).

Finally, although rarely reported, dietary phytoestrogens, particularly soy-derived compounds commonly found in commercial pet foods, have also been proposed as a potential source of estrogenic activity in spayed animals (Cerundolo et al. 2004).

Regardless of the source, elevated estrogen concentrations may result in estrous signs, bilateral symmetrical alopecia, vulvar edema, vulvar discharge, and, in severe cases, hematological abnormalities including pancytopenia secondary to bone marrow suppression.

## Clinical case

**Fig. 1:**
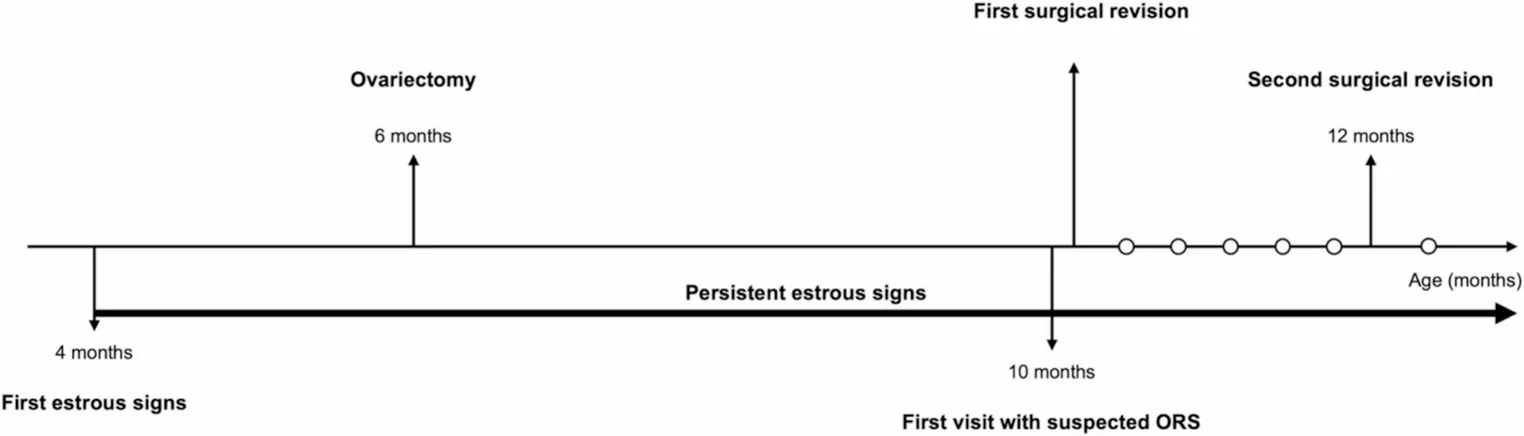
Clinical timeline of the case. Estrous signs first appeared at 4 months of age, and an ovariectomy was performed by the attending veterinarian at 6 months. At 10 months of age, the bitch was referred to the University Hospital for evaluation of suspected ovarian remnant syndrome (ORS). A few days later, the first surgical revision was performed by the attending veterinarian, followed by a second surgical revision at the University Hospital at 12 months of age. Open circles (○) indicate follow-up examinations performed at the University Hospital

A 10-month-old female Italian Greyhound was presented to the Reproduction Unit of the Small Animal Clinical Service, Department of Veterinary Medical Sciences, University of Bologna for evaluation of persistent vulvar edema and serosanguineous vulvar discharge. According to the owner, these clinical signs had first appeared at approximately 4 months of age, considerably earlier than the expected onset of puberty and estrous cycle in this breed. An additional atypical feature was the persistence of estrous signs, including vulvar swelling and vaginal discharge over the months without cyclical regression. The owner also reported that, from the onset of the first estrous signs, taking the bitch to the dog parks or other environments with multiple dogs had become increasingly difficult, due to the persistent marked attention from male dogs. At 6 months of age, because of the persistent and non-regressing clinical presentation, the attending veterinarian elected to perform an ovariectomy. However, no improvement in the clinical condition was observed postoperatively, and the signs persisted with similar intensity (Fig. 1). During the same period, the bitch also developed alopecia involving the dorsal region, limbs, and tail, which was initially attributed to suspected follicular dysplasia.

**Fig. 2:**
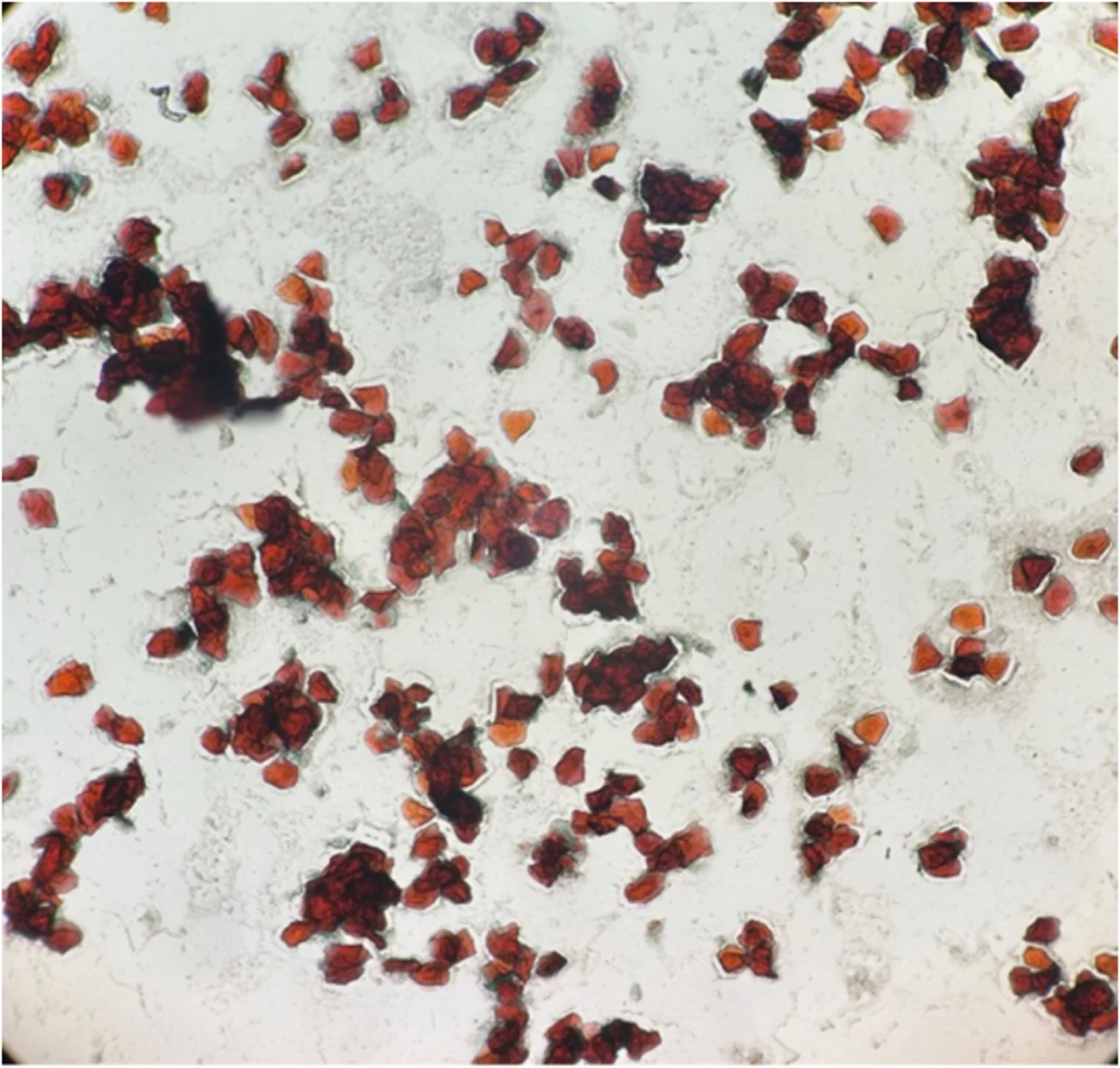
Vaginal cytology obtained at the initial clinical examination, showing a predominance of superficial cornified epithelial cells, consistent with estrogenic stimulation

At the initial presentation to the University Hospital, the owner was specifically asked about possible accidental exposure to exogenous hormonal sources; however, this was denied. A complete physical examination revealed marked vulvar edema and serosanguineous vulvar discharge, along with generalized alopecia. Abdominal ultrasonographic evaluation identified a rounded, anechoic structure in the region of the left ovarian pedicle. Vaginal cytology revealed a population composed of 100% superficial epithelial cells (Fig. 2), indicative of estrogenic stimulation, and serum progesterone concentration measured 1.99 ng/mL. Taken together, these findings supported a presumptive diagnosis of ovarian remnant syndrome (ORS). Moreover, the persistence of clinical signs over time suggested a pathological process affecting the remnant ovarian tissue, such as a cystic transformation or an endocrine-active neoplastic process (Zambelli et al. 2025).

Based on this diagnostic suspicion, the attending veterinarian performed a revision surgery the following week, as the owner elected to proceed with surgery at the same clinic for reasons of continuity of care and/or financial considerations. A hysterectomy was performed along with removal of the structure detected in the left ovarian region; however, no surgical excision was carried out in the right ovarian region because neither ultrasonographic nor macroscopic findings suggested the presence of remnant ovarian tissue at that site. Histopathological examination confirmed the excised tissue as functional ovarian tissue, without pathological abnormalities.

At the two-week postoperative follow-up examination, performed at the University Hospital, vulvar discharge had resolved; however, persistent vulvar edema and progression of alopecia were noted. Ultrasonography performed during the same visit revealed findings suggestive of postoperative tissue reaction in the left ovarian region, whereas vaginal cytology remained consistent with estrogenic stimulation, and progesterone levels were unchanged from previous measurements. These clinical and laboratory findings remained unchanged during the subsequent four follow-up visits at the same institution.

At this stage, two main differential diagnoses were considered. Despite the surgical revision, the first hypothesis involved the persistence of functional ovarian tissue, either normal or affected by pathological changes such as cystic degeneration or neoplasia, both potentially hormone-secreting. In the latter case, a neoplastic transformation of the left ovarian remnant could not be completely excluded despite the absence of histopathological evidence, as microscopic foci of pathological ovarian tissue may not be represented in the histological section examined. Consequently, the presence of metastatic lesions arising from such a neoplasm also had to be considered. The second hypothesis, assuming complete removal of ovarian remnant, was the presence of ectopic hormone-secreting tissue. This included lesions potentially representing metastases from the previously removed ovarian remnant, as well as primary hormone-secreting neoplasms located elsewhere in the body. However, greater emphasis was placed on the first hypothesis because the right ovarian region had not been inspected during the previous revision surgery. Therefore, a second exploratory laparotomy was performed at the University Hospital, including revision of both ovarian regions (right and left) and removal of residual scar tissue from previous surgeries, even in the absence of macroscopic abnormalities on either side. The excised tissue was then submitted for thorough histological examination, which confirmed the presence of an additional ovarian remnant in the right ovarian region containing corpora lutea. However, no evidence of tissue with estrogen-secretory activity was identified, making this finding unlikely to account for the persistent clinical signs. Consistent with this, three weeks after the second surgery, despite excision of this tissue, the bitch continued to exhibit marked vulvar edema, vaginal cytology consistent with estrogenic stimulation, and a progesterone concentration of 1.57 ng/mL.

Although the initial clinical history had been thoroughly collected, it later became evident that the owner herself had omitted crucial information: specifically, she was undergoing topical hormone replacement therapy containing estrogens and progestins. In particular, the estrogen-containing preparation was applied as a spray to the owner’s forearms. This represented a highly plausible source of exogenous hormonal exposure for the dog and could readily explain the persistence of estrogen-related clinical signs despite the most recent surgery. This hypothesis was further supported by the patient’s small size and close bond with the owner, as she spent a substantial amount of time being carried in her arms. In addition, progestin-based therapy was administered daily via vaginal suppositories. The continuation of progestin treatment was considered a plausible explanation for the persistence of non-basal progesterone concentration despite the surgical removal of ovarian tissue containing corpora lutea. Ideally, definitive diagnosis and optimal therapeutic management would have required complete prevention of exposure to these exogenous hormones. However, the owner reported that completely avoiding this source of exposure was initially not feasible.

**Fig. 3:**
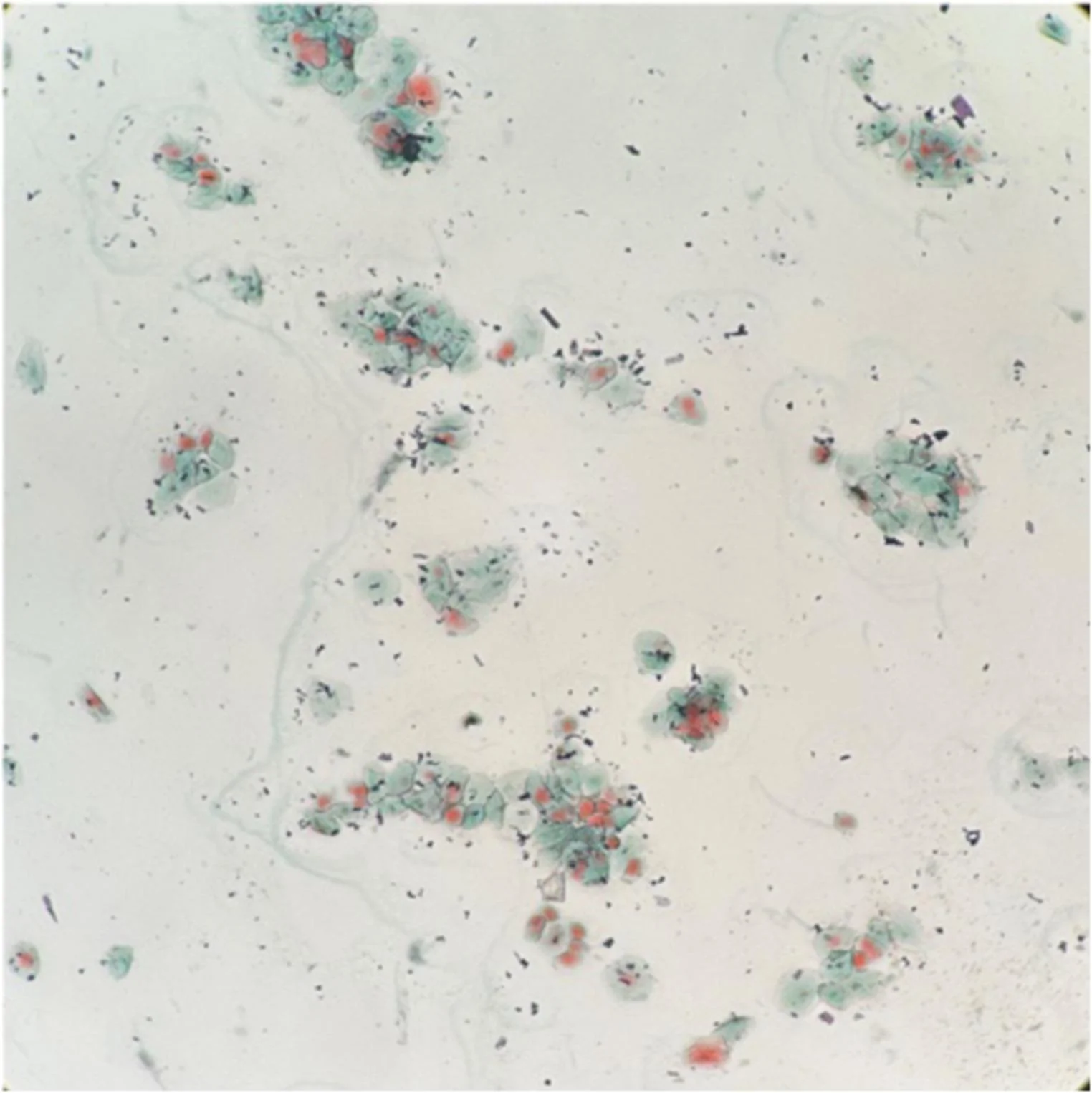
Vaginal cytology obtained 3 weeks after discontinuation of hormone therapy by the owner, consistent with the absence of estrogenic stimulation

As an alternative management approach, the owner decided to modify her routine by applying the hormonal therapy in the evening and limiting close contact with the dog during resting and sleeping periods. However, these adjustments did not result in any substantial clinical improvement in the patient. The owner therefore decided to discontinue her hormonal therapy completely. Three weeks after discontinuation, the first meaningful signs of improvement were observed: serum progesterone levels returned to basal values, and vaginal cytology no longer showed evidence of estrogenic stimulation (Fig. 3).

### Discussion and conclusions

In the case described, the findings support the view that ORS represents the most common, but not the only, cause of estrous signs in spayed bitches. Although anti-Müllerian hormone (AMH) assay has more recently been established as a valuable biomarker for diagnosing ORS (Place et al. 2011), diagnostic tools such as abdominal ultrasonography, vaginal cytology and serum progesterone measurement remain sufficient to support the diagnostic suspicion (Wallace 1991). In the present case, these standard evaluations were indeed adequate to establish the clinical suspicion and prompt the initial surgical revision, although the subsequent clinical course ultimately demonstrated that these findings required interpretation in the context of concurrent exposure to exogenous hormones.

However, to reliably exclude ORS as the cause of the clinical signs, meticulous surgical revision is essential, even in the absence of ultrasonographic or macroscopic evidence of residual ovarian tissue. This procedure should include a systematic exploration of both ovarian pedicles and the entire abdominal cavity, together with hysterectomy when the uterus is still present. Such an approach increases the likelihood of identifying even small ovarian remnants that may have been inadvertently displaced or overlooked during previous surgical procedures and still retain hormonal activity (Zambelli et al. 2025).

A meticulous histopathological examination of all excised tissue is strongly recommended to confirm the diagnosis and characterize the resected specimens (Zambelli et al. 2025). Furthermore, persistent residual ovarian tissue chronically exposed to elevated gonadotropin concentrations may be at increased risk of pathological changes, including neoplastic transformation (Buijtels et al. 2010).

Despite the surgical removal of two histologically confirmed ovarian remnants, estrous signs persisted in the present case, demonstrating that ORS alone could not fully explain the clinical picture. This finding highlights the need to consider additional causes capable of inducing estrogenic or persistent estro signs in spayed females.

Therefore, the correct interpretation of the clinical findings was only possible after obtaining an accurate and complete medical history. Although a thorough initial anamnesis had been performed, essential information was still missing because the owner had not reported certain details due to privacy concerns. This highlights a well-recognized limitation in clinical practice, whereby relevant information may be inadvertently omitted due to the owner’s perception of irrelevance or, in some cases, concerns regarding personal privacy. Similar patterns have been described in the literature, where incomplete or selectively reported histories can initially hinder a coherent understanding of the underlying condition and delay the diagnostic process (Berger et al. 2015; Sterman et al. 2019; Ganz and Wehrend 2021; Ivaldi et al. 2022; May et al. 2024). For this reason, particular emphasis should be placed on conducting a detailed and targeted anamnesis. The clinician should actively guide the interview through specific, structured questions to systematically explore all potential sources of exogenous hormonal exposure, environmental factors, and management-related practices that could contribute to the clinical presentation.

Once this additional information was obtained, the overall clinical picture became clear, allowing an appropriate interpretation of the patient’s persistent estrogenic signs.

## Data Availability

No datasets were generated or analysed during the current study.
